# Start from the end: Policy exploration to inform effective and consistent interventions applied to COVID-19 in St. Louis

**DOI:** 10.1093/pnasnexus/pgag155

**Published:** 2026-05-11

**Authors:** David O’Gara, Matt Kasman, Matthew D Haslam, Ross A Hammond

**Affiliations:** Division of Computational and Data Sciences, Washington University in St. Louis, One Brooking Drive, St. Louis, MO 63130, USA; Center on Social Dynamics and Policy, Brookings Institution, 1775 Massachusetts Ave NW, Washington, DC 20036, USA; Department of Health, City of St. Louis, 220 S. Jefferson Ave, St. Louis, MO 63103, USA; Division of Computational and Data Sciences, Washington University in St. Louis, One Brooking Drive, St. Louis, MO 63130, USA; Center on Social Dynamics and Policy, Brookings Institution, 1775 Massachusetts Ave NW, Washington, DC 20036, USA; School of Public Health, Washington University in St. Louis, One Brookings Drive, St. Louis, MO 63130, USA; Santa Fe Institute, 1399 Hyde Park Road, Santa Fe, NM 87501, USA

**Keywords:** agent-based modeling, emulators, epidemiology

## Abstract

Mathematical models are a powerful tool to study infectious disease dynamics and intervention strategies against them in social systems. However, due to their detailed implementation and steep computational requirements, practitioners and stakeholders are typically only able to explore a small subset of all possible intervention scenarios, a severe limitation when preparing for disease outbreaks. In this work, we propose a parameter exploration framework utilizing emulator models to make uncertainty-aware predictions of high-dimensional parameter spaces and identify large numbers of feasible response strategies. We apply our framework to a case study of a large-scale agent-based disease model of the COVID-19 “Omicron wave” in St. Louis, Missouri that took place from December 2021 to February 2022. We identify large numbers of response strategies that would have been estimated to have reduced disease spread by a substantial amount. We also identify policy interventions that would have been able to reduce the geospatial variation in disease spread, which has additional implications for designing thoughtful response strategies.

Significance statement Mathematical models have tremendous utility to inform decision-making in high-stakes scenarios: one recent application being the COVID-19 pandemic. However, due to their high computational cost, modelers and stakeholders are often limited in the amount of potential intervention strategies they may explore in a reasonable amount of time. In this work, we apply lightweight “emulators” (statistical approximations of complex simulators) to a large-scale COVID-19 model of St. Louis, Missouri. Our results show we are able to find a large amount of policy interventions estimated to reduce disease spread, with additional geospatial implications, offering a framework for rapid response planning in high-stakes decision making.

## Introduction

Epidemics of communicable disease have the capacity to exact a heavy toll on our society. Before, during, and after waves of epidemic spread, policymakers and stakeholders have used mathematical models of disease transmission to forecast potential scenarios, inform mitigation strategies, and explore how we can prepare for the next one (1–3). An important subset of available models are individual or “agent-based” models (ABMs), which explicitly simulate the interactions of individual actors in a population, offering opportunities to understand mechanisms of disease transmission and opportunities for intervention (4). These types of computational models have a long history of informing policy response planning for a multitude of diseases in the United States and worldwide (5–16).

A significant complication in application of this approach arises when policymakers and stakeholders must balance multiple competing objectives in evaluating potential responses: for example, not just limiting the total outbreak size but also minimizing economic and social disruption and remaining conscious of healthcare system capacity. This type of balancing act presents a steep challenge, which becomes even more difficult as more objectives are considered. Importantly, when assessing interventions in the face of multiple objectives, a globally optimal, one-size fits all solution likely does not exist (17, 18). ABMs have the potential to help policymakers explore what action to take, and understand trade-offs, substitutions, and limitations for any proposed policy interventions.

Reaching this potential requires overcoming technical hurdles. Due to the level of detail embedded in these models, the simulation and experimentation process can be quite expensive, sometimes requiring minutes or hours to complete a single model run (5, 9). Therefore, when faced with the question of how to intervene on a complex dynamical system of disease spread while simultaneously satisfying multiple objectives, modeling teams are limited by their computational “budget,” or the number of simulations they can conduct in a reasonable amount of time. These efforts are further complicated when epidemic and social dynamics depart from tidy canonical modeling assumptions such as an outbreak stemming from an index case in an otherwise completely susceptible population, complete and durable immunity conferred by vaccination, and perfect adherence to nonpharmaceutical interventions (19, 20). These assumptions and others did not apply to the late 2021 through early 2022 COVID-19 “Omicron wave” in the United States, resulting in the largest rates of infection over any similar time period (21–26). Because the Omicron wave happened after the initial stages of the pandemic, differential access to interventions and protective behaviors likely drove disparities in disease spread (27–31). Essentially, the Omicron wave represented a highly atypical phase of the epidemic, driven by high viral transmissibility and waning immunity, as well as changing attitudes regarding the palatability of nonpharmaceutical interventions in the population and thus, policies and intervention strategies that might have been effective for other phases or for previous epidemics may not have been effective during the Omicron wave. As a result, when planning for mitigation strategies, the question of what to do, as well as when and how to do it, becomes unclear, and the notion of a one-size fits all solution is likely not applicable in these circumstances. Complex adaptive systems models in general, and ABMs in particular, are well-suited to modeling scenarios when policy impacts are not uniform, with differential effects across subpopulations or settings (32–37).

Here, we introduce an approach to advance ABM’s capacity to explore the vast policy landscape of potential mitigation strategies, and illustrate the approach with an applied case study of a highly detailed ABM of COVID-19 spread in St. Louis, Missouri, the “TRACE-STL” model, which has already informed policy response planning in the greater metro area (38). Using available data, we parameterize TRACE-STL to look retrospectively at the Omicron wave and explore 10 different intervention strategies across vast implementation intensities, allowing us to investigate which mixtures of policies might have been effective in mitigating disease spread while considering multiple (potentially competing) objectives for outcomes. We do so via the use of emulator (or “surrogate”) models, a class of statistical models used to approximate expensive computer simulations, which can offer uncertainty-aware predictions at unseen model inputs, and guide model exploration across parameter space (18, 39).

A deep and mature literature for calibrating complex simulation models to available data exists, in which emulators have been used to great effect (15, 40–56). Some prior work has also investigated the use of emulators to identify optimal intervention strategies (57). The search for intervention strategies falls under the category of “model exploration,” a set of strategies to explore and characterize a computer model’s parameter space, of which model calibration (fitting a simulator model to data) and “model optimization” (identifying model parameters that yield an optimal policy response) are two examples (58). To our knowledge, the use of emulators to identify large regions of candidate policy configurations to inform tradeoffs, interactions, and decision-making has not yet been explored in great detail. Emulation for policies is additionally a fruitful approach because most emulator techniques (such as Gaussian processes [GPs], which we utilize here) may be trained sequentially or utilized for active learning (18). This offers a set of approaches to identify regions of parameter space where the simulator and emulator disagree, providing insight for the next batch of simulations to run to better adaptively represent the uncertain parameter space.

Our results indicate that thoughtful emulation is a powerful framework to explore the TRACE-STL model. The principle contribution of our framework is not to identify “optimal” policies, but to explore the myriad ways that interventions may affect complex epidemiological simulations, to quantify uncertainty across potential outcomes, and present a range of options to meet policy goals. We identify a large range of policy combinations, many of which are combinations of several lower intensity policies, which would be estimated to have reduced disease spread. We also study our framework’s ability to identify tradeoff and substitution effects for policies across wide ranges. Finally, we also demonstrate that our emulation strategy can not only lead to reductions in total disease spread but can also identify policies that are estimated to reduce the variation in disease burden across census tracts, offering support for the possibility of developing geospatially consistent interventions. Specifically, equalizing disease burden across census tracts or neighborhoods is of high importance to avoid concentrated social or economic costs, to prevent strain on local healthcare systems, and to prevent secondary epidemiological flare-ups. Taken together, our approach may be thought of as a detailed “response playbook” that could help policymakers plan and explore myriad epidemic response scenarios.

## Methods

### TRACE-STL model description

Our complex simulation setting is an updated version of the “TRACE-STL” ABM of COVID-19, parameterized and calibrated to the “Omicron wave” of disease spread from 2021 December 21 to 2022 February 19. TRACE-STL was originally developed to project robust policy response scenarios over a wide range of potential interventions and epidemiological scenarios (38). A full description may be found in the Supplementary material, but we recap the main components and model dynamics here. Briefly, TRACE-STL is a large-scale computational disease transmission model of 2.4 million agents in St. Louis, Missouri and its adjoining counties. The synthetic population and contact networks are based on the 2010 RTI Synthetic population data (59, 60). We use publicly available data on the number of cases, deaths, and tests (61–63) , mobility trends (64), vaccinations and boosting (65), and census-tract SVI (66) and simulate a 60-day period where each model timestep denotes one simulated day.

### Policy interventions

We simulate 10 different policy interventions, which are designed to reflect a wide range of potential policy scenarios. Each of the policies are varied over wide ranges, meant to reflect the status quo (at their lowest intensity) and a massive increase (at their highest intensity). Importantly, the policies affect the agent population in different ways: for example, a change in the frequency of diagnostic PCR testing is expected to have a different effect than administering vaccines. We note that we do not simulate social distancing policies, such as school closures and stay-at-home directives. Prior work with TRACE has explored the efficacy of such interventions (67). We conducted several exploratory simulations implementing wide-spread social distancing measures: for example, complete school closure reduced the number of cumulative infections to about one half of those observed under status quo conditions, and decreasing the average number of agent “community” contacts by 50% performed similarly, meaning that strictly according to reducing outbreak size, these interventions would have been effective. However, we assumed that implementing such policies at the required intensity would be infeasible, socially undesirable, or prohibitively expensive, and thus we did not vary them in our primary experiments. Thus, our policy explorations may be interpreted as potential alternatives to widespread social distancing measures. Our policy scenarios and ranges are reported in Table 1. We denote five of our policies as policies regarding changes in “coverage” ie the amount of an intervention available and applied (such as the number of vaccines) and five of our policies as changes in “behavior.” These designations are not meant to be completely descriptive or mutually exclusive but are given for ease of presentation and to demonstrate the many ways policies effect agent dynamics. For example, we note that an individual receiving a vaccine is likely a function of both product availability as well as individual behavior. Policy scenarios were chosen to represent potential options that may have been available to decision-makers during the study period as well as to define intervention strategies that affected agent behavior (and therefore model dynamics) in several different ways. These modifications to agent and model behavior are described in the rightmost column of Table 1.

**Table 1:** Policy descriptions and ranges in simulation experiments.

Policy	Description	Range	Adjusts model dynamics by
Coverage			
PCR tests per day	Multiplier from baseline	1x−10x	Increases number of agents who can be tested, contact traced and quarantined
Antigen tests per day	Multiplier from baseline	1x−10x	
Vaccines	Minimum % of each age group (5 and older) vaccinated	0–75% of each age group	Reduces transmission probability if infected, reduced infection probability if susceptible, subject to product-specific estimates of vaccine effectiveness and time-dependent immunity
Boosters	Minimum % of each age group (5 and older) boosted	0–50% of each age group	
Contact Tracing	Number of contacts traced per day	6,000–60,000	Increasing number of contacts who can be traced and asked to quarantine
Behavior			
Symptomatic testing OR	Odds ratio to test symptomatic agents (relative to nonsymptomatic)	10–100	Symptomatic agents are more likely to receive test, and have contacts traced
Testing quarantine OR	Odds ratio to test quarantined agents (relative to nonquarantined)	1–100	Quarantined agents are more likely to receive test (and have contacts be traced)
Quarantine adherence contact traced	Quarantine adherence when successfully contact traced	0.7–1.0	Increases probability of quarantining for full duration
Mask duration contact traced	When contact traced, wear a mask (days)	0–14	Increases duration of masking protection
Mask adherence	Multiplier on mask efficacy	0–0.2	Reduces transmission probability if infected, and reduces infection probability if susceptible

The range of each policy is presented such that the lower end of the range denotes our estimate of the policy intensity at baseline (meant to simulate observed conditions), and the upper end denotes a massive policy increase from baseline conditions. We note that for our vaccination and booster policies, the policies enact a minimum vaccination or booster proportion for each age group, but where empirical data exceeds the minimum, we use the amount given by data.

### Emulation of simulation outcomes

We simulate two model outcomes using TRACE-STL: the number of cumulative infections, reported at the model’s end, as well as the variance in cumulative infections stratified by the social vulnerability index (SVI). Specifically, we calculate the attack rate (number of infections divided by population size) for each SVI category: 0–0.25, 0.25–0.5, 0.5–0.75, and 0.75–1.0, and take the variance. Given a model outcome *Y* and a dataset *X* that is n rows×p columns (p=10), our emulation method is a GP. Specifically, we assume the data generation process:


(1) $$Y_{i}∼N(m_{i}(X),K_{\theta }(X,X)),$$

where $m_{i}(X)$ denotes a mean function, and $K_{\theta }$ is our covariance matrix, denoting the correlation of our model inputs with one another. In the case of our primary outcome (cumulative infections), since the model outcome is highly nonlinear and varies across a wide range, we first model the mean response with a gradient-boosting machine (GBM), and then fit a heteroskedastic GP regression to the residual $Y_{1}-{\hat{Y}}_{1}^{\mathrm{GBM}}∼N(0,K_{\theta }(X,X))$. Thus, for an unobserved input X’, our prediction is:


(2) $${\hat{Y}}_{1}=\mathrm{GBM}(X^{\prime })+\mu_{\mathrm{GP}}(X^{\prime }),$$

where $\mu_{\mathrm{GP}}(X^{\prime })$ is the mean prediction under a GP conditional on observed data (39, 68, 69).

In the case of the secondary outcome (SVI variance), we assume a zero-mean function. For both outcomes, we use a Matérn ν=5/2 kernel for the covariance and estimate hyperparameters via maximum likelihood.

### Generating variation in infection rates by census tract

Throughout the COVID-19 pandemic, the disease burden has fallen unevenly across socio-economic strata, with more socially vulnerable communities being at higher risk of infection (28, 30, 31, 70, 71) . Contemporary work has postulated that this due at least in part to more vulnerable groups being less able to restrict their mobility (such as remote work, social distancing, and quarantine) (72). Since we do not use individual-level data of infections, we use the following model mechanism to generate heterogeneity in infection risk by SVI. At model instantiation, each agent is given a probability that they will quarantine if they are symptomatic or test positive for COVID-19. This probability is calculated as:


(3) $$\text{Pr}(\text{quar})=0.5\;*\;\text{Pr}(\text{quar\_adhere})+0.5\;*\;(1.0-{\text{SVI}}_{\mathrm{tract}}),$$

where ${\text{SVI}}_{\mathrm{tract}}$ is the SVI in an agent’s home census tract, meaning that agents in more vulnerable tracts (closer to 1.0) will be less likely or able to quarantine than agents in less vulnerable tracts, and quar_adhere is the population level quarantine probability. Importantly, in the context of policy implementation, this also means that a quarantine-focused policy would have to provide social support and resources to quarantine individuals, as this mechanism explicitly accounts for an agent’s broader social context rather than quarantine compliance simply being about individual choice, a distinction made clear in prior work and is shown again here (13, 38). We also note that this construction is sensitive to the baseline quarantine adherence probability, and thus depending on the model parameterization, the distribution of per-agent quarantine probabilities may not span the full range of 0 to 1. For example, if the baseline quarantine adherence is 0, then the per-agent quarantine probability would be bound between 0 and 0.5, and if the baseline quarantine adherence is 1, then the per-agent quarantine probability would be bound between 0.5 and 1.

### Sensitivity analysis

We analyze the sensitivity of several of our core model parameters to calibration targets, namely: the base transmission rate, presymptomatic proportion, multiplier on initial infections, symptomatic testing odds ratio, and quarantine adherence. These parameters were deemed the most uncertain based on available literature as well as our prior work with the TRACE model (38, 67). We selected the chosen model parameterizations via manual exploration and testing while building the newest version of TRACE-STL over a period of several months and several hundred simulation experiments. However, we also note that calibration of a model to empirical data is often a labor-intensive task, meaning that modeling teams must balance their total “simulation budget” (ie limitations imposed by computational and personnel resources as well as consideration of timely findings) between calibration as well as studying interventions. These sensitivity analysis results are available in Figs. S1–S3. We also report the induced distribution of secondary infections as a function of our base transmission rate (the mean of which corresponds to $R_{0}$ in a fully susceptible population with a single index case) in Fig. S4.

### Computational implementation

We used TRACE-STL version 2.0.0 for the simulations in this work. TRACE-STL is programmed in Python 3.11. For our emulators, we used hetGPy version 1.0.2 (69) for our heteroskedastic GPs, and LightGBM via optuna version 4.2.0 (73) for our GBM. Our GBM was trained on the mean cumulative infection response across 20 replicates and hyperparameters were selected via 10-fold cross-validation.

## Results

### Generating a status quo model of the Omicron wave

**Figure 1:**
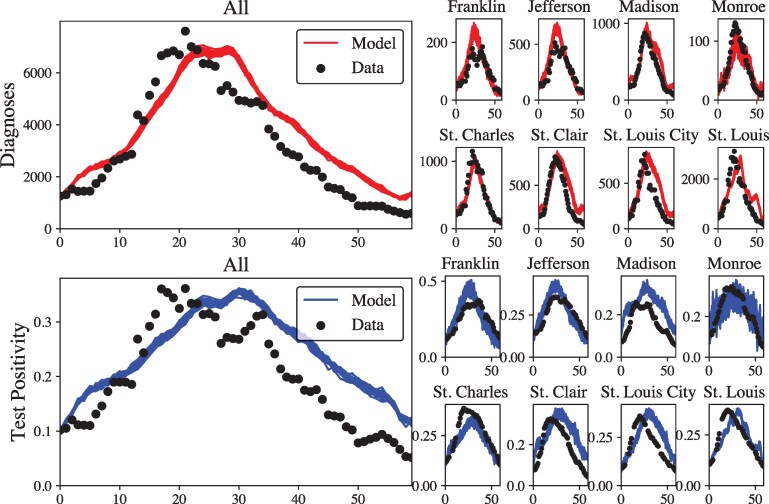
Calibration of model parameters to available data. The top panels show the number of diagnoses per simulated day, at both the population (“All”) and county levels. Points represent the 7-day average of cases per day, and lines represent model output. The bottom panels show the number of positive tests divided by number of tests reported, at both population and county levels. The model parameters that were varied included the base transmission rate, the number of initially infected agents, and the odds ratio for symptomatic agents to take a test. Their ranges are given in the Supplementary material.

We establish three primary calibration targets for the Omicron wave at two scales of analysis, namely the number of confirmed cases over time, the test positivity rate, and estimated underreporting of cases relative to total infections at the county and aggregate level. In Figure 1, we are able to recover the trend of confirmed cases as well as the test positivity rate across counties and at the population level. We also find that the model reports an average of 842,356 (SD 3,733) cumulative infections and 225,044 confirmed cases (SD 848) compared to 191,759 cases based on CDC data. Our model ratio of 1 in 3.74 cases being reported (confirmed cases divided by infections) is consistent with contemporary estimates during the Omicron wave (74–76). This is driven by the proportion of asymptomatic cases, test availability, and the imperfect likelihood that symptomatic agents will be tested. We find that model stochasticity across populations and random seeds is somewhat low, due to the model’s large number of initially infected agents, as opposed to simulating an outbreak via an index case, which would lead to much higher stochasticity. However, this was intentional in our analysis, because having a large pool of initially infected agents is harder to disrupt, and shifts the focus to mitigation, rather than containment. At baseline, we also find that the mean SVI-stratified cumulative infection rate is 40.53% (SD 0.27) for the most socially vulnerable census tracts (SVI between 0.75 and 1.0, 106 census tracts with 333,593 agents) to 32.35% (SD 0.18) for the least socially vulnerable tracts (between 0 and 0.25, 265 census tracts total with 1,043,650 agents).

### Singular policy interventions would be estimated to reduce disease spread, but would require large increases from baseline

Our first experiment explores the efficacy of varying our 10 policy interventions over a Latin hypercube sample of size n=1,500 (drawn from a sample of 1,489 locations augmented with baseline conditions, and 10 additional parameterizations corresponding to the highest intensity of each policy intervention and all other policies held to baseline) with 20 replicates at each design location for a total of 30,000 simulations. A single simulation of TRACE-STL takes about 2 min on a high-performance computing cluster, meaning the initial set of experiments corresponded to about 1,000 h of CPU time. As discussed in Methods, we train a GBM on the mean response of cumulative infections and fit a heteroskedastic GP regression to the residuals. We also used a second Latin hypercube sample of 500 simulations to assess the global fit of the emulator on parameterizations it was not trained on as a validation set, as well as a secondary set of validation simulations varying one policy at a time. For the primary validation simulations, the root mean squared error (RMSE) between simulator and emulator output is 14,265.71 compared to the training simulator vs. emulator RMSE of 5,086.25, which is a substantial improvement over a naive linear model fit (training RMSE=30,676.99, validation RMSE=29,381.33). A comparison of training vs. primary validation points is given in Fig. S5. We also explore two other analyses in our construction of the initial design and emulation strategy: first, we assess the emulation strategy by instead training a neural network instead of a GP regression. We also assess the efficiency of the design space: while a large Latin hypercube sample is the standard in the computer experiments literature (39) due to its space-filling properties, recent work (77) has explored acquiring samples via integrated mean-squared prediction error (IMSPE), an approach we adopt here for half of the simulations (and the other half are acquired via a space-filling design). We experimented with several neural architectures, but found that the neural network’s performance on the validation set was worse than our original emulator (training RMSE=117,947.64 , validation RMSE=106,738.51. We also note that tuning neural architectures to a given simulator context is an open problem in the literature (78), and while GP regression comes with its own set of challenges, it generally requires fewer hyperparameters and manual tuning compared to neural networks (18, 39, 79). For the IMSPE sequential design strategy, the global fit was also worse than our original emulator (training RMSE=100,401.30, validation RMSE 230,416.94) and we also note that the sequential design strategy greatly increased the wall-time of running the simulations, since for the space-filling design, the simulations could be run in an embarrassingly parallel format, indicating that a space-filling design is more appropriate in this case.

**Figure 2:**
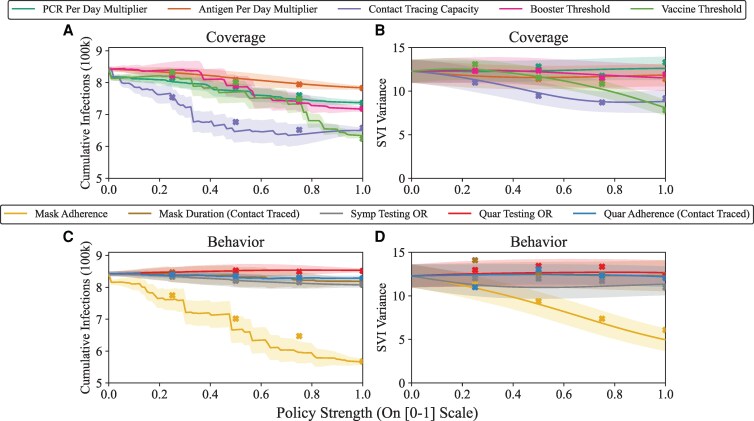
Varying singular policy interventions. Each of our 10 policies are varied from their lowest intensity (meant to estimate the policy configuration at baseline) to their highest (representing a substantial increase from baseline) and are estimated via emulators. Our 10 policies are split into two categories in the top and bottom panels: “Coverage” policies are denoted in (A) and (B) and “Behavior” policies are denoted in (C) and (D) as discussed in Methods. *Y* axes represent the cumulative infections over a model run (left panels) and the variance in SVI-stratified disease spread (right panels). For both outcomes, lower values indicate a more favorable outcome (leading to less infection, as well as less variation in disease spread as measured by SVI). Shaded regions represent 90% predictive intervals, and *x*’s denote validation runs (simulations of policies not explicitly included in the training set with the exception of the points representing the highest policy strength along the right-hand edge of each panel).

In Figure 2, we assess the estimated efficacy of each of our emulated policy interventions by varying them to their lowest (status quo) to highest (representing a substantial increase) while holding all other policies at their status quo values. We find that some policy interventions (especially, contact tracing capacity, mask adherence, and vaccine threshold) reduced estimated disease spread (in some cases below 700,000 cumulative infections, and <600,000 for mask adherence), but would require large increases from the status quo to accomplish this. We also find that for the emulated interventions shown, a vaccine-focused policy tends to outperform a booster-focused policy if only one can be varied. This comports with a priori expectations since the ceiling for vaccination is higher (a minimum of 75% of all age groups, as opposed to 50% for boosters, which were chosen as modeling assumptions for the minimum vaccination rate for each age group), as well as the potential size of the eligible boosted population being constrained by vaccination in the first place, as well as having a sufficient amount of time since vaccination. For example, we find that setting a target of boosting 50% of the population but maintaining status quo vaccination numbers results in 39.7% (SD 0.02) of the population receiving boosters. Finally, we find that none of the policies alone, save for mask adherence, would be expected to reduce the variation in SVI-stratified disease spread. We also generally find our emulated estimates of cumulative infections and SVI-stratified variance are in agreement with simulator outputs, as can be seen in by the secondary validation simulations marked as “x’s” in the figure, which follow the trend as policy strength increases and mostly fall within the predictive intervals. Each line corresponds to simulating a grid of 100 predicted points, and had we used the same number of replicates (20 per parameterization) this would have corresponded to 20⋅100⋅10=20,000 additional simulations, or about 667 h of CPU time. With the use of emulators, we were able to make these predictions in a matter of seconds.

### Combining interventions for interaction effects

**Figure 3:**
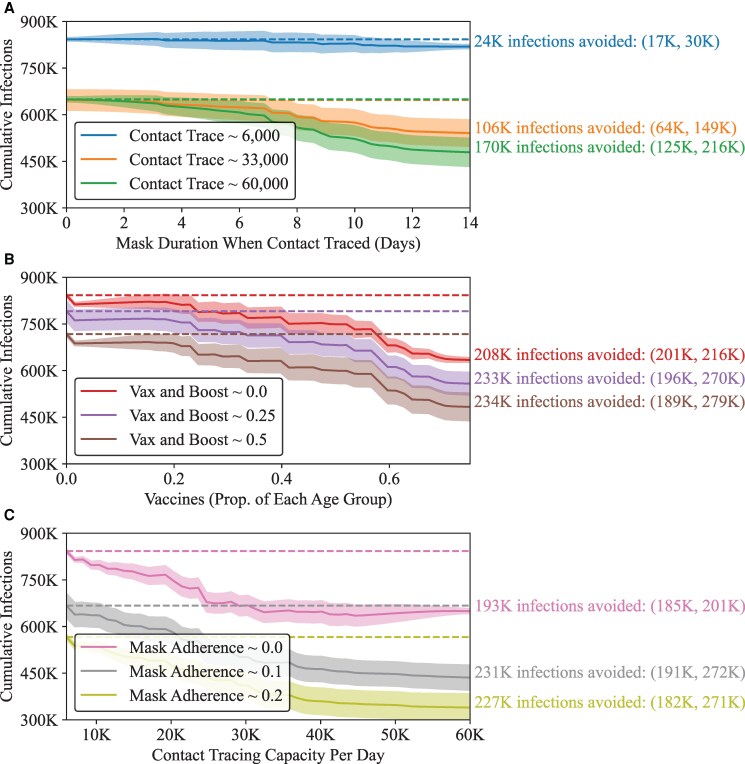
Investigating trade-offs and substitutions when combining policies. In all panels, the *Y*-axis denotes the cumulative number of infections over a model run (note the truncation at 300 K infections). The *X*-axis in each panel varies a policy from its baseline value through its full policy range. We show three combinations of policies across the panels: contact tracing and masking duration when contact traced (A), vaccination and boosting (B); and contact tracing and mask adherence (C). Dashed lines represent the cumulative infections for the affected policy and holding all other policies to baseline. Text on the right-hand side of each plot reports the difference in infections holding the *X*-axis policy at baseline and at its highest intensity for each of the three levels of the policies represented by dashed lines. Shaded regions and text in parentheses represent 90% predictive intervals. See Table 1 for policy descriptions.

The efficacy of several of our policy interventions may be improved when other interventions are present at appropriate intensities. In particular, since our “mask duration when contact traced” has agents wear a mask when they are reached by contact tracing, we anticipate that the with increased contact tracing, we expect the efficacy of that policy to increase. In Fig. 3, we examine three such scenarios. Figure 3A shows the effect of asking agents to wear masks for a certain number of days after being contact traced (from 0 to 14 days) for contact tracing capacities of 6,000, 33,000, and 60,000 agents per day. We can see that the marginal effect increasing “mask duration when contact traced” from 0 to 14 days results in an estimated 24,000 infections being avoided at the baseline value of 6,000 for tracing capacity, but 170,000 infections avoided when there is a substantial increase in contact tracing, since the contact tracing process initiates masking in agents who are at a higher risk of being infected, disrupting potential transmission chains. Similarly, Fig. 3B demonstrates the interplay between initial vaccine availability supporting the efficacy of a booster-focused policy: timely prevaccination of at least 75% of eligible age groups and a booster target of 50% of those in eligible age groups results in an estimated 234,000 infections being avoided, compared to a booster-focused policy alone, reducing the outbreak size to about 483,000 cumulative infections (an over 42% decrease from baseline). The emulation process also underscores the opportunities to uncover potential tradeoffs and thresholds, which are shown when vaccination increases from baseline (in red) can substitute for booster doses (in purple) such as in the range of 40% (where the lines cross). Figure 3C showcases the interaction of the two most effective nonpharmaceutical interventions. We emphasize here that the policy combinations and interactions shown represent only a small slice of the 10D policy parameter space. Due to the nonlinear behavior of the individual policies, as well as their interactions with one another, it is difficult to characterize the global performance of the policies via simple measures (as can be done with a simpler model such as linear regression coefficients), and thus it is helpful to think of the emulators as “prediction machines” as is becoming common practice with the interpretation of statistical models in the sciences (80). For further exploration and investigation, our simulation data, emulators, and analysis code are all publicly available (81).

### Pooling policies demonstrates a large landscape of low-intensity policies to mitigate spread

**Figure 4:**
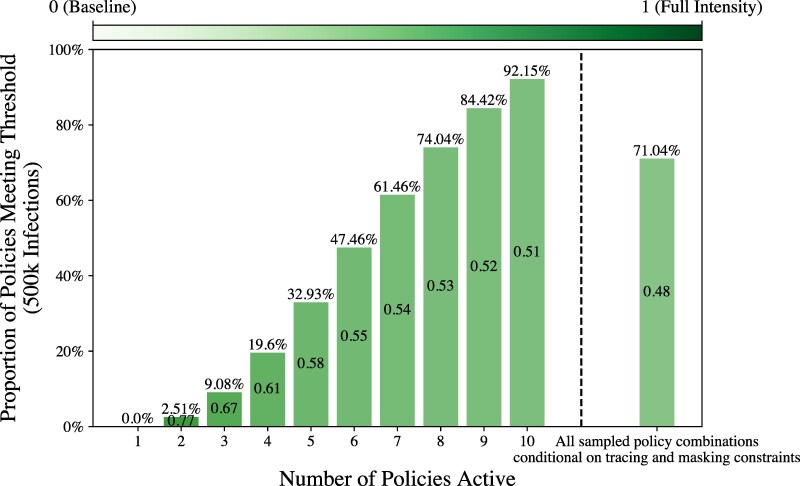
Pooling mixtures of many low-intensity policies estimated to meet policy goals. The *x*-axis shows the number of policies “active” at one time (set to a higher value than baseline), and the *y*-axis shows the percentage of emulated policies estimated to meet the policy goal of 500,000 cumulative infections or less. Each bar is shaded according to the average policy intensity (0 representing baseline, and 1 representing the largest policy value) of the active policy combinations. For k=10 (in the unconstrained case), we emulate 500,000 potential policy combinations. The rightmost bar represents the scenarios where all 10 policies were varied, but only consider candidate interventions with no more than 33,000 contact traced per day and mask adherence no more than 0.1 (half of the range explored for each policy). All other bars contain the results of 5,000*(10k) policy combination emulations where *k* is the number of active policies.

Prior work, both for COVID-19 transmission as well as other communicable diseases has demonstrated that singular policy interventions are often outperformed by combining multiple interventions, especially ones that affect the population in differential ways (9, 10, 13). However, the strength of these different interventions must be prespecified a priori, and when policy options are numerous, modelers are restricted to only testing a few values of each intervention. With our emulation strategy, we can explore far more potential intervention scenarios than would be feasible to simulate with the TRACE-STL model. This allows us to specify model outcomes of interest and search for intervention scenarios that satisfy this criteria. As a motivating example, we investigate the efficacy of the number of interventions deployed at one time, as well as the required intensity of those interventions to meet a policy goal: in this case, no more than 500,000 cumulative infections among the roughly 2.4 million agents. These results are shown in Fig. 4, where we sequentially increase the number of “active” policies (with intensities higher than their baseline values) and calculate the percentage of estimated policies that meet the policy goal. Specifically, we do the following: For each number of active policies k∈[1,…,9], we take a Latin Hypercube sample of 5,000 for each combination of (10k) policies and fix the remaining policies to baseline. We also calculate the average policy intensity of the active policies. Finally, for k=10, we draw a sample of 500,000 candidate policy combinations to emulate a massive landscape of potential intervention strategies. As mentioned previously, with an estimated two minutes per simulation run, simulating these 500,000 options would have corresponded to nearly 17,000 h (or 2 years) of CPU time but were able to be emulated on a laptop in a small fraction of the time, about eight minutes. We see that by increasing the number of policies, we are able to simultaneously identify more candidate policies to meet the policy goal, and that combinations of lower intensity mixtures may be identified. As a sensitivity and robustness check, we also explore the policy combinations that utilize <33,000 contacts traced per day and a mask adherence of <0.1, each of which were the midpoint of the full range of those policies, since they are individually effective, but increasing them to their full intensity may not be as feasible or cost-effective compared to milder increases of other policies. The percentage of these policy combinations meeting the policy goal is 71.04%. Limiting the two most effective policies represents just one choice of potential logistical, social, or economic constraint to designing and discovering policy combinations, but one could easily study performance under different constraints using our available emulated data (81).

### Policy specification can reduce disease spread while improving geospatial consistency in outcomes

We validate our policy findings by selecting the 10 lowest-intensity policies satisfying both our policy goal and the self-imposed constraint (<500,000 infections, no policy using more than the midpoint of contact tracing capacity and mask adherence) and simulating them with TRACE-STL. We select the 10 “smallest” (lowest intensity) policies by ranking them according to the norm of the policy vector:


(4) $$\text{policy combination intensity}=\sqrt{\sum_{i=1}^{10}p_{i}^{2}},$$

where $p_{i}$ represents each individual policy strength on a [0-1] scale, and $p_{i}=0\;\forall i$ corresponds to baseline. Note that this differs slightly from the previous analysis where we reported the mean policy intensity: $\frac{1}{k}\sum_{i=1}^{k}p_{i}$ where $p_{i}>0$. This was chosen as a preference for policy combinations that had a mixture of multiple policies (for a 2D example, this is equivalent to saying that combining two policies at half strength would be preferable to doing one at full strength, ie that $0.5^{2}+0.5^{2}=0.5\leq 0^{2}+1^{2}=1$).

**Figure 5:**
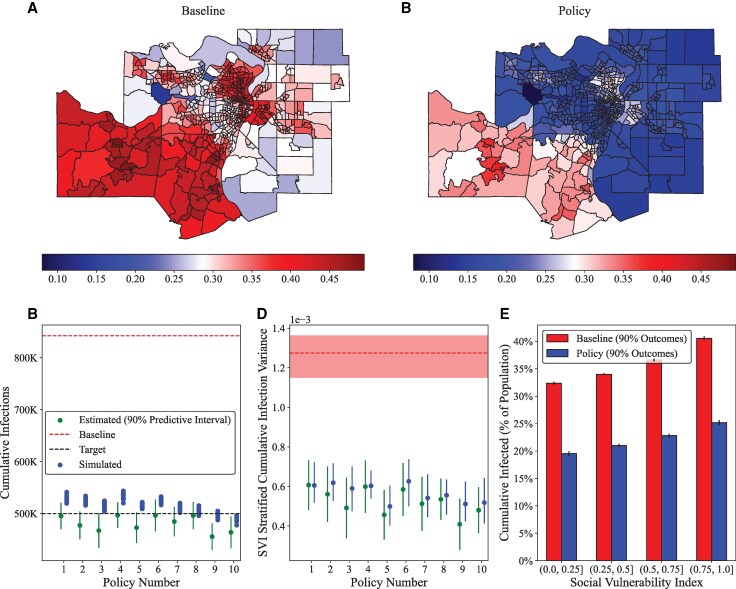
Policy emulation comports with simulation outcomes and can identify geospatially consistent policies. A) Census-tract level disease spread at baseline, where color denotes the proportion of agents in each census tract (measured by home location) infected during a model run. B) The same calculation under a policy counterfactual mixing many low-intensity policies together, which achieves substantially lower disease spread as well as lower variance across SVI categories. Our 10 simulated policy mixtures are shown in (C) and (D), reporting results for both cumulative infection as well as the variance SVI-stratified disease spread. E) The cumulative infection rates by SVI category at baseline and under the policy counterfactual. Error bars in (C) and (D) reflect the 90% predictive intervals from the emulators.

In Fig. 5, we demonstrate that we are able to systematically predict the cumulative infection rate using our emulation strategy compared with those from the model while reducing the variance in SVI-stratified disease spread (Fig. 5C). Figure 5A shows cumulative disease spread by census tract at baseline, while Fig. 5B shows cumulative disease spread under one of our simulated policy combinations (specifically the one corresponding to “Policy Number 3” in Fig. 5C and D). The specific policy implementations are available in Table S1, and notably use a relatively high value of contact tracing capacity (∼28,000 contacts per day) supported by a somewhat higher mask duration when contact traced (∼3 days) and higher testing availability (∼2.1× for PCR, ∼2.4× for antigen) which allow the contact tracing process to identify more individuals and break the chain of infection for agents who are successfully traced. Since our measure of social vulnerability is based on agent geographic location, we expect a policy that acts on dynamic information, such as contact tracing, to play a role in reducing geospatially stratified disease spread. Figure 5E shows the difference in SVI-stratified disease spread between baseline and policy #3, notably in the reduction for the most socially vulnerable agents. In fact, the variance in SVI-stratified disease spread at baseline ($1.27\times 10^{-3}$) is more than twice as high as under our policy combination ($5.69\times 10^{-4}$). While in some cases our emulated predictions predict a lower total of cumulative infections than are reflected by the simulations, we also note that we intentionally selected the ten lowest intensity policy combinations estimated to meet the criteria of 500,000 cumulative infections, meaning that these are among the most challenging policies to meet the criteria, or can be thought of as approximating a minimum threshold for policy combinations, since increasing any one policy in the policy combination is expected to reduce the number of cumulative infections, as was shown in Figs. 2 and 3 (eg more vaccination or more contact tracing leading to fewer infections). However, an important consideration is whether these proposed policies using the emulator actually improve upon our initial set of 30,000 simulations from the space-filling design. In our case, they do: each of the 10 proposed policies are strictly nondominated by the other 1,500 policies from the initial set of simulations, when measured by policy norm and cumulative infections. In other words, none of the original policies are both smaller in norm and result in fewer cumulative infections, meaning that the 10 proposed policies improve the Pareto efficient frontier. These policies are visualized along their norm and cumulative infection axes in Fig. S6.

The choice of preferring many low-intensity policy combinations instead of large singular increases is also just one option among several candidate strategies. For example, other work has prioritized selecting outcomes that differ from one another (58) which in our case would correspond to preventing candidate policy combinations from overlapping with one another. As an additional analysis, we select a random sample of policy combinations estimated to yield between 475,000 and 500,000 cumulative infections (in the range of the policies) via a maximin design to offer insight into many effective policies that are maximally different from one another. Those policies are available in Table S2 and visualized in Fig. S7. This analysis also underscores the importance of choosing how to characterize policy combination preferences, any of which could also be investigated in our available emulated data (81). Finally, as the simulator-to-emulator fidelity is most critical in the region of parameter space corresponding to our outcome of interest (in this case, around 500,000 cumulative infections and low-intensity policy combinations) we select an additional 500 samples from this region and simulate with TRACE-STL to validate the fidelity of our predictions. The RMSE between simulated and emulated cumulative infections across these 500 samples is 17,233.53, and the rank correlation coefficient is 0.409,(P<0.001), a substantial improvement over predictions with a linear model (RMSE=28,184.72, rank correlation = 0.260,(P<0.001)). If increased simulator-to-emulator fidelity is required, we also performed an additional experiment where we altered the objective function: instead of emulating cumulative infections, we emulated the objective $f(x)=(Y-5{)}^{2}+\|x{\|}_{2}$ via a heteroskedastic GP, where *Y* is the cumulative number of infections (divided by 100,000), 5 is the desired target of 500,000 infections, and the norm of *x* penalizes the size of the policy vector. Since minimizing this objective directly promotes our desired policy combinations, we acquire 200 additional simulations selected via the expected improvement active learning criterion (18). With these additional 200 simulations, the RMSE between simulated and emulated outcome is 6,392.50 and the rank-correlation coefficient is 0.93 (P<0.001). These 200 simulations are also themselves highly performant, resulting in a average cumulative infection of around 508,000 (492,000–522,000 in the 25–75% quantiles) and an average policy norm of 1.24, compared to the original batch of simulations which only had 870 simulations (<3% of the batch) in a similar range with an average policy norm of 1.59. The key observation here is that we may supplement a *global* emulator (ie trained on the full design space) with acquisitions to support a *local* goal (acquiring low-intensity policies near a desired outbreak size target). Though we are encouraged by the high rate of agreement between simulated and emulated outcomes, we also emphasize that our goal with emulation is not to rank the proposed policies against one another, but that the goal of the analysis was to use the emulator to query the space of interventions for policies that might meet the policy goal, and then use the simulator to ascertain whether they actually would, and which in these experiments, did.

## Discussion

When preparing for disease response, policymakers typically have a short time to act and must choose from several potential intervention scenarios in the face of potentially competing outcome objectives, high complexity, and significant cost. Detailed mechanistic simulation models can help explore interventions and identify the most effective way to intervene given objectives and constraints, but extensive experimentation is often infeasible except for the simplest models. In this work, we have demonstrated the utility of emulators as a tool to significantly expand the ability of computational models to explore intervention scenarios in complex situations. We show how thoughtful use of emulation can predict simulator outputs at untested input locations, resulting in a more detailed exploration of parameter space, with high potential utility for policymakers, since it would allow decision makers to “start from the end” and specify their outcome objectives ahead of experimentation (such as in our case of filtering to interventions resulting in <500,000 infections) and then systematically solve for intervention scenarios that achieve this outcome. We accomplish by using standard tools from the computer experiments and Bayesian optimization literatures, but extended beyond their typical use cases such as calibrating a model to empirical data or solving for a single “optimal” intervention, but instead solving for a collection of policies that are expected to meet a desired outcome and are more feasible than larger, singular interventions. We also highlight some of the challenges and pitfalls with this framework: namely that it requires a global emulator of simulator parameter space (though it can be augmented with specific observations from a local area of parameter space to accommodate specific analyses), it requires sufficient uncertainty quantification to ensure that simulator-to-emulator fidelity is preserved in regions of policy interest, and proper specification of what a “good” policy is (in our two cases, policy size and across-policy discrepancy) in order to be useful for decision makers. Additionally, an auxiliary benefit of the use of emulators for scenario exploration is the further democratization of exploratory policy modeling, as decision makers such as local and state public health agencies may not have the computational resources to run expensive simulation models, but may have the resources to utilize emulators, which can typically run on a laptop.

However, we do note that this “policy target” analysis has the benefit of hindsight in the case of the COVID-19 Omicron wave, and choosing an outbreak threshold or outcome before it happens may bring additional challenges in practice. To combat these difficulties, our emulation framework naturally allows for the exploration of tradeoffs, substitutions, and sensitivity analysis, all of which are critical pieces of information a policymaker would want to have in a high-stakes scenario. In our experiment, there were a multitude of low-level policy combinations that met the objective. These findings may be advantageous for policymakers who wish to meet their goals while implementing policies that are tailored to their specific community, especially in cases where community receptiveness to policies varies by context, such as masking and vaccination coverage during the COVID-19 pandemic. Finally, our results here underscore that identifying suitable interventions for complex disease dynamics is still a challenging problem, and even mixtures of many policy interventions that were identified as “small” relative to our full search space of model parameterizations still represent somewhat large increases from the status quo observed in data, and underlines the need for policymakers to have substantial resources at their disposal to mitigate disease spread. As mentioned earlier, our focus on identifying the smallest necessary increases from baseline that meet the disease reduction target and reduce variation in geographic disease spread likely have downstream benefits in that they may be less socially disruptive and more cost-effective, and more likely to prevent secondary epidemiological flareups, offering evidence that they may be more efficient than other candidate sets of policy interventions. We also note that our choice of studying the Omicron wave of COVID-19 spread in St. Louis was motivated by the atypical nature of the Omicron wave, and thus, the set of policy combinations identified may not be applicable to other locations or even other phases of the epidemic. This is also in part due to the nonstationary dynamics of disease spread, population behavior, and the implementation of the policies themselves. For example, a similar study calibrated to the Delta variant (the Summer and Fall of 2021) would have had to at a minimum contend with lower vaccine coverage and fewer boosters (65), but higher pharmaceutical efficacy (82), as well as differences in population mobility and viral transmission (62). Thus, one would not immediately expect the same performance of policy interventions as during the Omicron wave. However, we believe the framework summarized in this work, specifically the use of emulators to explore policy parameter space along with careful selection of outcome measures, may be useful to modelers and decision-makers.

This work could be extended in several ways. We focused on the specific application of a detailed COVID-19 simulation model, but the methods and framework are likely applicable to other types of complex simulation models used for scientific inquiry and to inform policy choices. Of particular interest is further exploration of multiobjective optimization, especially when the objectives are in opposition. Using our epidemiological model as an example, a natural extension would be to model competing objectives such as cumulative disease spread and economic cost, political feasibility, or social disruption (with the model incorporating appropriate assumptions about how these change alongside increases in policy intensity). Recent work has explored the use of emulators in this context (17), and extensive exploration of high-dimensional multiobjective complex simulation models represents a promising research direction. Finally, the use of active learning techniques to sequentially sample the space of candidate interventions and efficiently identify suitable parameterizations depending on the policy goal should be further investigated in future work.

## Supplementary Material

pgag155_Supplementary_Data

## Data Availability

The data and source code underlying this article are archived via Zenodo at https://doi.org/10.5281/zenodo.15993040. Source code of the TRACE-STL model is available at https://github.com/davidogara/csdp-trace-stl.

## Preprints

A preprint of this article is published at https://doi.org/10.48550/arXiv.2507.10870.

## References

[1] Epstein JM . 2009. Modelling to contain pandemics. Nature. 460:687–687. 19661897 10.1038/460687a PMC3785367

[2] Howerton E, et al 2023. Evaluation of the US COVID-19 scenario modeling hub for informing pandemic response under uncertainty. Nat Commun. 14:7260. 37985664 10.1038/s41467-023-42680-x PMC10661184

[3] Loo SL, et al 2024. The US COVID-19 and influenza scenario modeling hubs: delivering long-term projections to guide policy. Epidemics. 46:100738. 38184954 10.1016/j.epidem.2023.100738 PMC12444780

[4] Hammond RA . Overview of current concepts and process for agent-based modeling. In: *New horizons in modeling and simulation for social epidemiology and public health*. John Wiley and Sons Inc, 2021. p. 31–43.

[5] Aylett-Bullock J, et al 2021. June: open-source individual-based epidemiology simulation. R Soc Open Sci. 8:210506. 34295529 10.1098/rsos.210506 PMC8261230

[6] Eubank S, et al 2004. Modelling disease outbreaks in realistic urban social networks. Nature. 429:180–184. 15141212 10.1038/nature02541

[7] Ferguson N, et al 2020 Report 9: impact of non-pharmaceutical interventions (NPIs) to reduce COVID19 mortality and healthcare demand. Technical Report, Imperial College London.

[8] Ferguson NM, et al 2006. Strategies for mitigating an influenza pandemic. Nature. 442:448–452. 16642006 10.1038/nature04795 PMC7095311

[9] Germann TC, Kadau K, Longini IM, Macken CA. 2006. Mitigation strategies for pandemic influenza in the United States. Proc Natl Acad Sci U S A. 103:5935–5940. 16585506 10.1073/pnas.0601266103 PMC1458676

[10] Halloran MElizabeth, et al 2008. Modeling targeted layered containment of an influenza pandemic in the United States. Proc Natl Acad Sci U S A. 105:4639–4644. 18332436 10.1073/pnas.0706849105 PMC2290797

[11] Hinch R, et al 2021. OpenABM-Covid19—an agent-based model for non-pharmaceutical interventions against COVID-19 including contact tracing. PLoS Comput Biol. 17:e1009146. 34252083 10.1371/journal.pcbi.1009146 PMC8328312

[12] Hladish TJ, et al 2016. Projected impact of dengue vaccination in Yucatán, Mexico. PLoS Negl Trop Dis. 10:e0004661. 27227883 10.1371/journal.pntd.0004661 PMC4882069

[13] Kerr CC, et al 2021. Controlling COVID-19 via test-trace-quarantine. Nat Commun. 12:2993. 34017008 10.1038/s41467-021-23276-9 PMC8137690

[14] Longini IM, et al 2005. Containing pandemic influenza at the source. Science. 309:1083–1087. 16079251 10.1126/science.1115717

[15] Ozik J, Wozniak JM, Collier N, Macal CM, Binois M. 2021. A population data-driven workflow for COVID-19 modeling and learning. Int J High Perform Comput Appl. 35:483–499.

[16] Penny MA, et al 2016. Public health impact and cost-effectiveness of the RTS, S/AS01 malaria vaccine: a systematic comparison of predictions from four mathematical models. Lancet. 387:367–375. 26549466 10.1016/S0140-6736(15)00725-4 PMC4723722

[17] Binois M, Collier N, Ozik J. 2025. A portfolio approach to massively parallel Bayesian optimization. J Artif Intell Res. 82:137–167. 41000331 10.1613/jair.1.16868 PMC12459664

[18] Garnett R . Bayesian optimization. Cambridge University Press, 2023.

[19] Hunter E, Mac Namee B, Kelleher JD. 2017. A taxonomy for agent-based models in human infectious disease epidemiology. J Artif Soc Soc Simul. 20:2.

[20] Shastry V, Reeves DCale, Willems N, Rai V. 2022. Policy and behavioral response to shock events: an agent-based model of the effectiveness and equity of policy design features. PLoS One. 17:e0262172. 35061776 10.1371/journal.pone.0262172 PMC8782474

[21] Badillo-Goicoechea E, et al 2021. Global trends and predictors of face mask usage during the COVID-19 pandemic. BMC Public Health. 21:2099. 34781917 10.1186/s12889-021-12175-9 PMC8667772

[22] Fridman I, Lucas N, Henke D, Zigler CK. 2020. Association between public knowledge about COVID-19, trust in information sources, and adherence to social distancing: cross-sectional survey. JMIR Public Health and Surveill. 6:e22060. 10.2196/22060 PMC7511226 32930670

[23] King WC, Rubinstein M, Reinhart A, Mejia R. 2021. Time trends, factors associated with, and reasons for COVID-19 vaccine hesitancy: a massive online survey of US adults from January-May 2021. PLoS One. 16:e0260731. 34932583 10.1371/journal.pone.0260731 PMC8691631

[24] Pro G, et al 2021. US trends in mask wearing during the COVID-19 pandemic depend on rurality. Rural Remote Health. 21:6596. 34252284 10.22605/RRH6596 PMC8827623

[25] Troiano G, Nardi A. 2021. Vaccine hesitancy in the era of COVID-19. Public Health. 194:245–251. 33965796 10.1016/j.puhe.2021.02.025 PMC7931735

[26] Yan Y, et al 2021. Measuring voluntary and policy-induced social distancing behavior during the COVID-19 pandemic. Proc Natl Acad Sci U S A. 118:e2008814118. 33820846 10.1073/pnas.2008814118 PMC8076999

[27] Faust JS, et al 2024. Racial and ethnic disparities in age-specific all-cause mortality during the COVID-19 pandemic. JAMA Netw Open. 7:e2438918. 39392630 10.1001/jamanetworkopen.2024.38918 PMC11581672

[28] Kim SJ, Bostwick W. 2020. Social vulnerability and racial inequality in COVID-19 deaths in Chicago. Health Edu Behav. 47:509–513. 10.1177/1090198120929677 PMC8183499 32436405

[29] Lundberg DJ, et al 2023. COVID-19 mortality by race and ethnicity in us metropolitan and nonmetropolitan areas, March 2020 to February 2022. JAMA Netw Open. 6:e2311098. 37129894 10.1001/jamanetworkopen.2023.11098 PMC10155069

[30] Mody A, et al 2022. Quantifying inequities in COVID-19 vaccine distribution over time by social vulnerability, race and ethnicity, and location: a population-level analysis in St. Louis and Kansas City, Missouri. PLoS Med. 19:e1004048. 36026527 10.1371/journal.pmed.1004048 PMC9417193

[31] Oates GR, et al 2021. The association between neighborhood social vulnerability and COVID-19 testing, positivity, and incidence in Alabama and Louisiana. J Commun Health. 46:1115–1123. 10.1007/s10900-021-00998-x PMC8106900 33966116

[32] Combs TB, et al 2019. Modelling the impact of menthol sales restrictions and retailer density reduction policies: insights from tobacco town Minnesota. Tob Control. 29:502–509. 31462580 10.1136/tobaccocontrol-2019-054986 PMC7476266

[33] Hammond RA, et al 2020. Development of a computational modeling laboratory for examining tobacco control policies: tobacco town. Health Place. 61:102256. 32329725 10.1016/j.healthplace.2019.102256 PMC11410381

[34] Kasman M, et al 2023. Childhood sugar-sweetened beverage consumption: an agent-based model of context-specific reduction efforts. Am J Prev Med. 65:1003–1014. 37451323 10.1016/j.amepre.2023.07.004 PMC10787028

[35] Kasman M, Kreuger LKurt. Best practices for systems science research. National Institutes of Health Office of Behavioral and Social Sciences Research, 2022.

[36] The Human Factor . 2024 Anticipating pitfalls in AI application to healthcare. Technical Report, Brookings Institution.

[37] Luke DA, et al 2017. Tobacco town: computational modeling of policy options to reduce tobacco retailer density. Am J Public Health. 107:740–746. 28398792 10.2105/AJPH.2017.303685 PMC5388950

[38] Hammond R, Ornstein JT, Purcell R, Haslam MD, Kasman M. 2021 Modeling robustness of COVID-19 containment policies.

[39] Gramacy RB . Surrogates: Gaussian process modeling, design and optimization for the applied sciences. Chapman Hall/CRC, 2020.

[40] Fadikar A, et al 2018. Calibrating a stochastic, agent-based model using quantile-based emulation. SIAM/ASA J Uncertain Quantif. 6:1685–1706.

[41] Farah M, Birrell P, Conti S, De Angelis D. 2014. Bayesian emulation and calibration of a dynamic epidemic model for A/H1N1 influenza. J Am Stat Assoc. 109:1398–1411.

[42] Katzfuss M, Guinness J, Lawrence E. 2022. Scaled Vecchia approximation for fast computer-model emulation. SIAM/ASA J Uncertain Quantif. 10:537–554.

[43] Kennedy MC, O’Hagan A. 2001. Bayesian calibration of computer models. J R Stat Soc B: Stat Methodol. 63:425–464.

[44] Krouglova Anastasia N, Johnson Hayden R, Confavreux Basile, Deistler Michael, Gonçalves Pedro J. 2025 Multifidelity simulation-based inference for computationally expensive simulators [preprint]. arXiv, arXiv:2502.08416, https://arxiv.org/abs/2502.08416.

[45] Langmüller AM, et al 2025. Gaussian process emulation for exploring complex infectious disease models. PLoS Comput Biol. 21:e1013849. 41460880 10.1371/journal.pcbi.1013849 PMC12774377

[46] Matveeva A, Leonenko V. 2022. Application of Gaussian process regression as a surrogate modeling method to assess the dynamics of COVID-19 propagation. Procedia Comput Sci. 212:340–347. 36437869 10.1016/j.procs.2022.11.018 PMC9682405

[47] O’Gara David, Kerr Cliff C, Klein Daniel J, Binois Mickaël, Garnett Roman, Hammond Ross A. 2024. Improving policy-oriented agent-based modeling with history matching: a case study [preprint]. arXiv, arXiv:2501.00616, https://arxiv.org/abs/2501.00616. 10.1016/j.epidem.2025.100845 40695109

[48] Panovska-Griffiths J, et al 2023 Machine learning assisted calibration of stochastic agent-based models for pandemic outbreak analysis. preprint, In Review.

[49] Pokharel G, Deardon R. 2016. Gaussian process emulators for spatial individual-level models of infectious disease. Can J Stat. 44:480–501.

[50] Reiker T, et al 2021. Emulator-based Bayesian optimization for efficient multi-objective calibration of an individual-based model of malaria. Nat Commun. 12:7212. 34893600 10.1038/s41467-021-27486-z PMC8664949

[51] Sauer A, Gramacy RB, Higdon D. 2023. Active learning for deep Gaussian process surrogates. Technometrics. 65:4–18.

[52] Sawe SJ, et al 2024. Gaussian process emulation to improve efficiency of computationally intensive multidisease models: a practical tutorial with adaptable R code. BMC Med Res Methodol. 24:26. 38281017 10.1186/s12874-024-02149-x PMC10821551

[53] Shattock AJ, et al 2022. Impact of vaccination and non-pharmaceutical interventions on SARS-CoV-2 dynamics in Switzerland. Epidemics. 38:100535. 34923396 10.1016/j.epidem.2021.100535 PMC8669952

[54] Trostle P, Guinness J, Reich BJ. 2024. A Gaussian-process approximation to a spatial sir process using moment closures and emulators. Biometrics. 80:ujae068. 39036985 10.1093/biomtc/ujae068 PMC11261348

[55] Vernon I, et al 2022. Bayesian emulation and history matching of JUNE. Philos Trans R Soc A: Math Phys Eng Sci. 380:20220039. 10.1098/rsta.2022.0039 PMC9376712 35965471

[56] Vernon I, Goldstein M, Bower RG. 2010. Galaxy formation: a Bayesian uncertainty analysis. Bayesian Anal. 5:619–669.

[57] Chandak A, Dey D, Mukhoty B, Kar P. 2020. Epidemiologically and socio-economically optimal policies via Bayesian optimization. Trans Indian Natl Acad Eng. 5:117–127. 38624421 10.1007/s41403-020-00142-6 PMC7333587

[58] Ozik J, Collier NT, Wozniak JM, Macal CM, An G. 2018. Extreme-scale dynamic exploration of a distributed agent-based model with the EMEWS framework. IEEE Trans Comput Soc Syst. 5:884–895. 30349868 10.1109/TCSS.2018.2859189 PMC6195352

[59] RTI International . 2010 U.S. Synthetic Population Ver. 1. Available at: https://github.com/RTIInternational/SyntheticPopulations/tree/main.

[60] Wheaton WD, et al 2009. Synthesized population databases: a US geospatial database for agent-based models. Methods Rep (RTI Press). 2009:905. 20505787 10.3768/rtipress.2009.mr.0010.0905 PMC2875687

[61] USA Facts . US COVID-19 cases and deaths by state, January 2020. Section: coronavirus.

[62] Reinhart A, et al 2021. An open repository of real-time COVID-19 indicators. Proc Natl Acad Sci U S A. 118:e2111452118. 34903654 10.1073/pnas.2111452118 PMC8713778

[63] Johns Hopkins University . Govex/COVID-19: data analysis and visualizations of daily COVID cases report.

[64] Google . COVID-19 community mobility reports.

[65] Centers for Disease Control and Prevention . COVID-19 vaccinations in the United States, County | Data | Centers for disease control and prevention.

[66] CDC . SVI interactive map, 2024.

[67] O’Gara D, et al 2023. TRACE-Omicron: policy counterfactuals to inform mitigation of COVID-19 spread in the United States. Adv Theory Simul. 6:2300147. 38283383 10.1002/adts.202300147 PMC10812885

[68] Binois M, Gramacy RB, Ludkovski M. 2018. Practical heteroscedastic Gaussian process modeling for large simulation experiments. J Comput Graph Stat. 27:808–821.

[69] O’Gara D, Binois M, Garnett R, Hammond RA. 2025. hetGPy: heteroskedastic Gaussian process modeling in Python. J Open Source Softw. 10:7518.

[70] Dasgupta S, et al 2020. Association between social vulnerability and a county’s risk for becoming a COVID-19 hotspot—United States, June 1–July 25, 2020. MMWR. 69:1535–1541. 33090977 10.15585/mmwr.mm6942a3 PMC7583500

[71] Lopes R, et al 2024. Combining genomic data and infection estimates to characterize the complex dynamics of SARS-CoV-2 Omicron variants in the US. Cell Rep. 43:114451. 38970788 10.1016/j.celrep.2024.114451

[72] Chang S, et al 2021. Mobility network models of COVID-19 explain inequities and inform reopening. Nature. 589:82–87. 33171481 10.1038/s41586-020-2923-3

[73] Akiba T, Sano S, Yanase T, Ohta T, Koyama M Optuna: a next-generation hyperparameter optimization framework. In: *Proceedings of the 25th ACM SIGKDD International Conference on Knowledge Discovery & Data Mining, KDD ’19*. Association for Computing Machinery; 2019. p. 2623–2631.

[74] Institute for Health Metrics and Evaluation . IHME COVID-19 projections, 2020. Publication Title: Institute for Health Metrics and Evaluation.

[75] Millimet DL, Parmeter CF. 2022. COVID-19 severity: a new approach to quantifying global cases and deaths. J R Stat Soc A: Stat Soc. 185:1178–1215. 10.1111/rssa.12826 PMC9115431 35600509

[76] Wang Y, Kumbhakar SC. 2025. COVID-19 under-reporting: spillovers and stringent containment strategies of global cases. J Product Anal. 63:87–106.

[77] Binois M, Huang J, Gramacy RB, Ludkovski M. 2019. Replication or exploration? Sequential design for stochastic simulation experiments. Technometrics. 61:7–23.

[78] Cranmer K, Brehmer J, Louppe G. 2020. The frontier of simulation-based inference. Proc Natl Acad Sci U S A. 117:30055–30062. 32471948 10.1073/pnas.1912789117 PMC7720103

[79] Christopher KI Rasmussen W, Edward C. Gaussian processes for machine learning. Vol. 2. MIT Press, 2006.

[80] Arel-Bundock V . Model to meaning: how to interpret statistical models with R and Python. CRC Press, 2025.

[81] O’Gara D . 2025 Archive of trace-STL version 2.0 and experiments.

[82] Menegale F, et al 2023. Evaluation of waning of SARS-CoV-2 vaccine-induced immunity: a systematic review and meta-analysis. JAMA Netw Open. 6:e2310650. 37133863 10.1001/jamanetworkopen.2023.10650 PMC10157431

