# Supplementary material for: Start from the end: Policy exploration to inform effective and consistent interventions applied to COVID-19 in St. Louis
Source: PNAS Nexus. 2026 May 11;5(5):pgag155. doi: 10.1093/pnasnexus/pgag155 (PMC13195302; doi:10.1093/pnasnexus/pgag155)
Supplement: pgag155_Supplementary_Data [file pgag155_supplementary_data.pdf]

PNAS nexus

Supplementary Information for

Start from the End: Policy Exploration to Inform Effective and Consistent Interventions applied to COVID-19 in St. Louis

David O'Gara, Matt Kasman, Matthew D. Haslam, and Ross A. Hammond

David O'Gara
Email: david.ogara@wustl.edu

This PDF file includes:

Figures S1 to S6
Table S1 to S3

Other supplementary materials for this manuscript include the following:

TRACE-STL Model Description.pdf

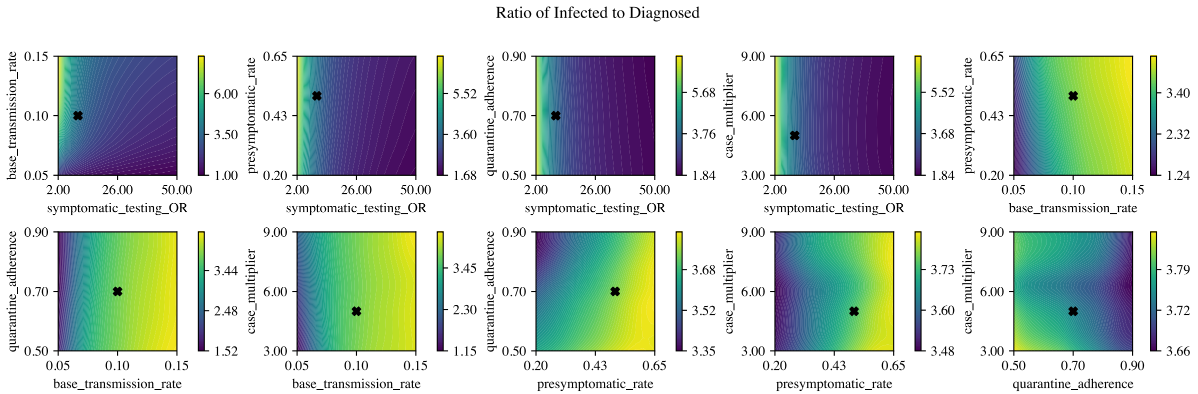

Fig. S1. Sensitivity analysis of core model parameters for ratio of infections to diagnoses. Each panel varies two model parameters from their baseline values (marked in “x” on each panel). Panels are shaded according to their expected ratio of infections to diagnoses (holding all other model parameters to their baseline values) based on an emulator fit of 500 samples from a Latin Hypercube and 10 model replicates at each parameterization.

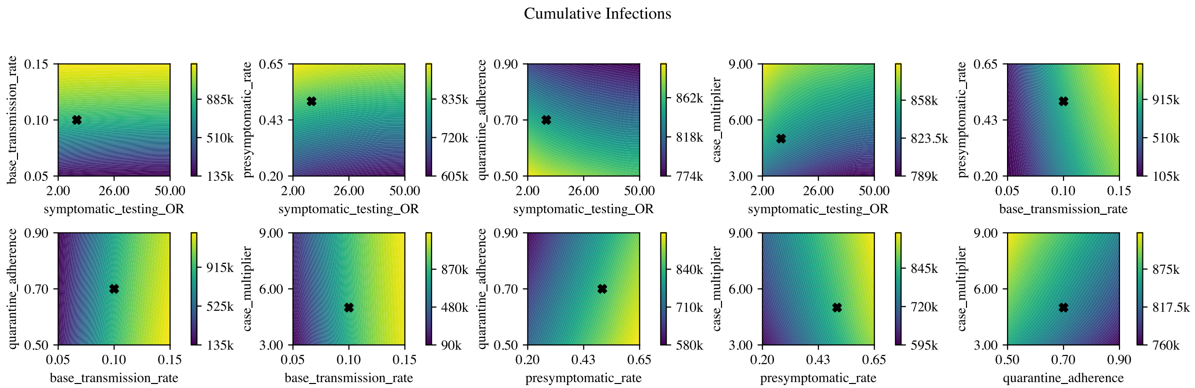

Fig. S2. Sensitivity analysis of core model parameters for cumulative infections. Each panel varies two model parameters from their baseline values (marked in "x" on each panel). Panels are shaded according to their expected cumulative infections (holding all other model parameters to their baseline values) based on an emulator fit of 500 samples from a Latin Hypercube and 10 model replicates at each parameterization.

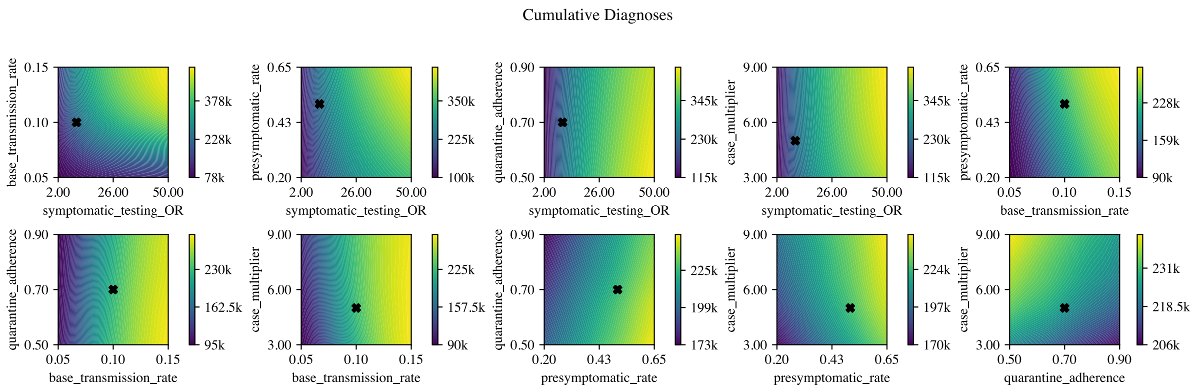

Fig. S3. Sensitivity analysis of core model parameters for cumulative diagnoses. Each panel varies two model parameters from their baseline values (marked in "x" on each panel). Panels are shaded according to their expected cumulative diagnoses (holding all other model parameters to their baseline values) based on an emulator fit of 500 samples from a Latin Hypercube and 10 model replicates at each parameterization.

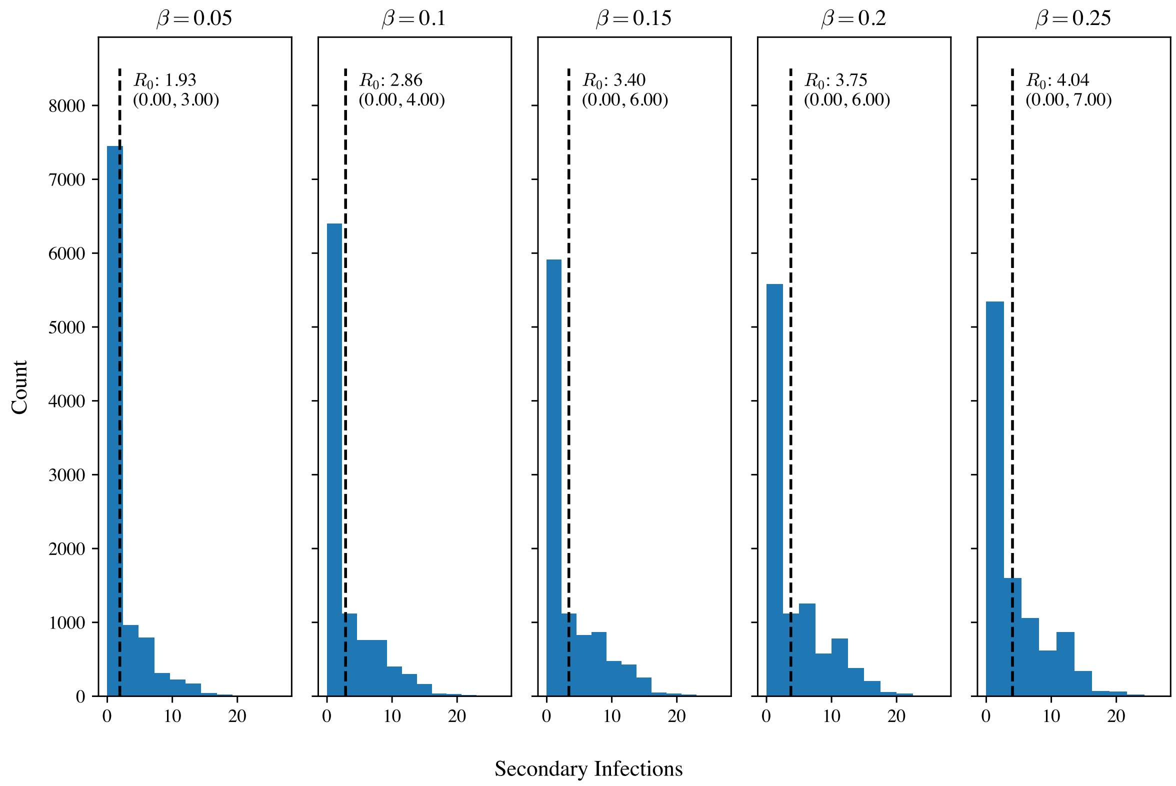

Fig. S4. R_0 calibration for base transmission rate. Each panel shows a value for the average base transmission rate and 10,000 model simulations of an index case in an otherwise susceptible population. The x-axis reports the number of secondary infections induced by the base transmission rate. Panel labels show the average number of secondary infections, and the middle 50% (25th and 75th percentile) of secondary infections in parentheses.

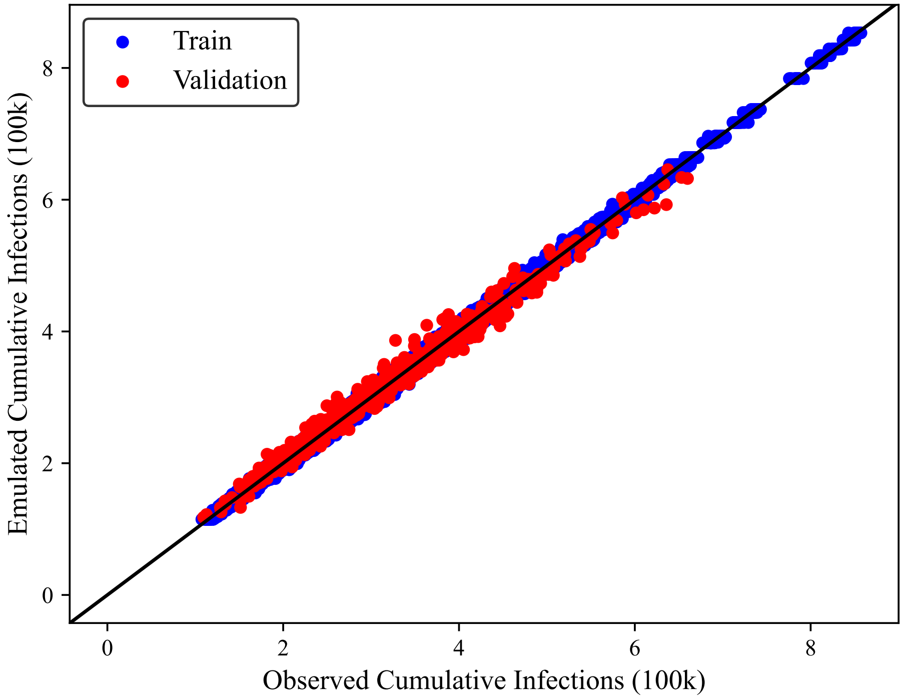

Fig. S5. Emulator validation data vs training data. Validation samples were selected from a Latin Hypercube sample of size 500. The x-axis represents the observed number of cumulative infections (from simulator output) and the y-axis represents the emulated number of cumulative infections (from emulator

output). The black line represents a hypothetical perfect agreement between emulator and simulator. Both the training samples and validation samples generally fall along the black line, indicating no substantial disagreement between simulator and emulator output across model parameter space.

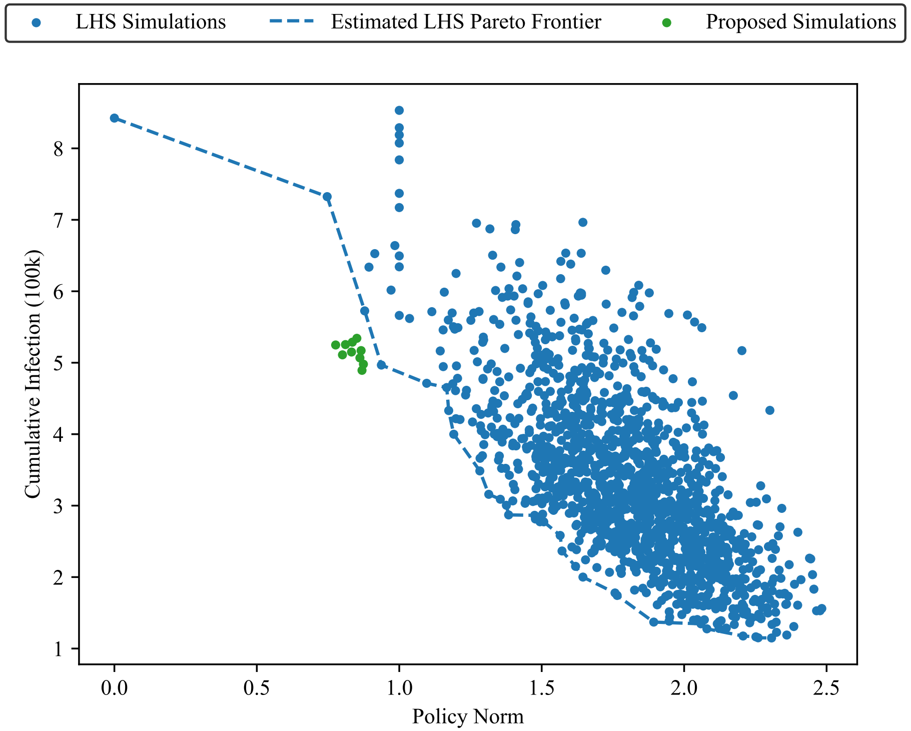

Fig. S6. Proposed policies expand the Pareto frontier beyond the original simulations. The x-axis represents the norm of the policy vector on the 0-1 scale, and the y-axis represents the number of cumulative infections during a model run. The proposed simulated policies discussed in Figure 5 of the main text were selected due to being near the policy target of 500k infections and being small in norm. Each of the ten proposed policies are strictly non-dominated by the original policies proposed in the Latin hypercube sample (LHS) simulations. Stated more simply, none of the original policies are both smaller in policy size and lead to fewer cumulative infections.

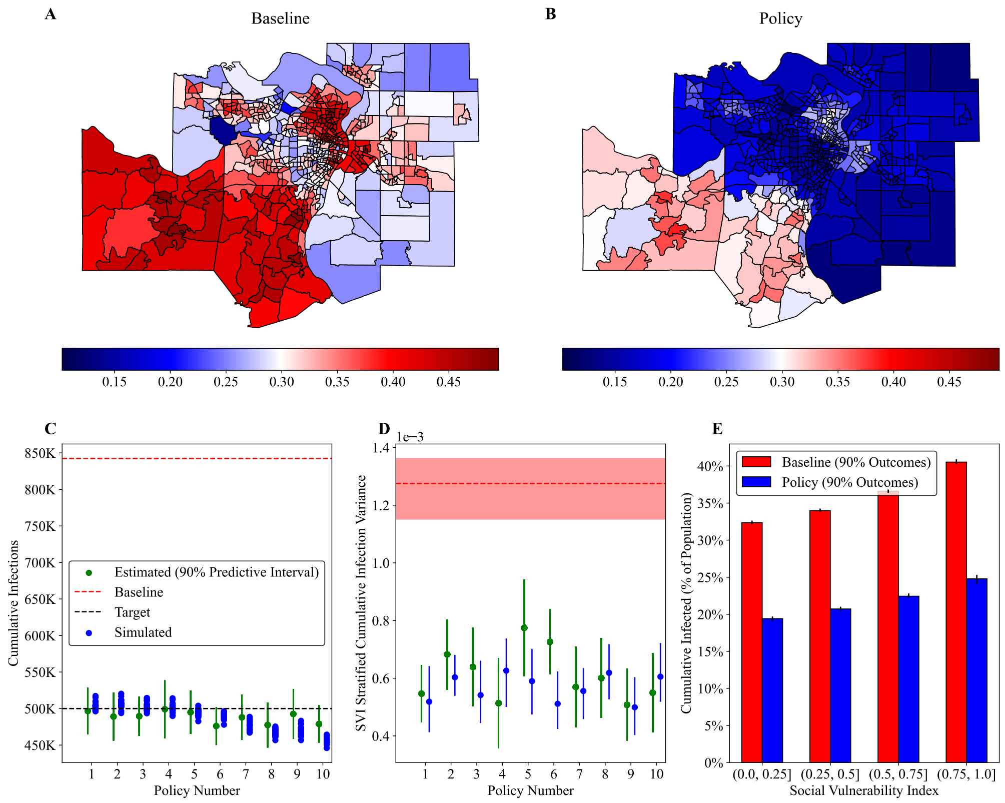

Fig. S7. Policy emulation comports with simulation outcomes and can identify geospatially consistent policies. Policy samples were selected via a maximin design of policies estimated to yield between 475,000 and 500,000 cumulative infections. Panel (A) shows census-tract level disease spread at baseline, where color denotes the proportion of agents in each census tract (measured by home location) infected during a model run. Panel (B) shows the same calculation under a policy counterfactual of one of the selected policies (#3), which achieves substantially lower disease spread as well as lower variance across SVI categories. Our ten simulated policy mixtures are shown in panels (C) and (D), reporting results for both cumulative infection as well as the variance SVI-stratified disease spread. Panel (E) reports the cumulative infection rates by SVI category at baseline and under the policy counterfactual. Error bars in panels (C) and (D) reflect the 90% predictive intervals from the emulators.

Table S1. Policy values for geospatially consistent policies. Table values show the specific policy parameterizations for the 10 "lowest intensity" policy combinations estimated to meet the policy goal of 500,000 cumulative infections or less. Policy descriptions are available in Table 1 in the main text.

Index	PCR Per Day Multiplier	Antigen Per Day Multiplier	Contact Tracing Capacity	Booster Threshold	Vaccine Threshold	Mask Adherence	Mask Duration (Contact Traced)	Symp Testing OR	Quar Testing OR	Quar Adherence (Contact Traced)
1	2.01	2.86	27,854	0.1	0.27	0.08	2	15.56	16.21	0.7
2	1.78	2.92	30,809	0.14	0.05	0.07	4	27.9	7.4	0.73
3	2.16	1.45	22,641	0.25	0	0.09	2	12.8	5.81	0.78
4	1.15	1.79	28,274	0.07	0.07	0.09	3	26.55	14.25	0.81
5	2.66	4.14	28,294	0.02	0.21	0.06	3	29.39	19.71	0.76
6	1.39	4.62	23,869	0.18	0.16	0.07	3	32.69	6.87	0.75
7	3.88	1.31	26,112	0.1	0.03	0.07	4	36.11	8.23	0.8
8	1.33	1.25	29,677	0.16	0.02	0.1	0	12.54	28.95	0.82
9	3.72	1.96	32,096	0.07	0.02	0.09	0	17.05	21.45	0.81
10	1.18	1.69	29,974	0.17	0.13	0.1	3	32.62	24.5	0.74
Range	[1,10]	[1,10]	[6k,60k]	[0.0,0.5]	[0.0,0.75]	[0,0.2]	[0,14]	[10,100]	[1,100]	[0.7,0.9]

Table S2. Policy values for geospatially consistent policies. Table values show the specific policy parameterizations for 10 policies selected via a “maximin” design so policies are spread out from one another in model parameter space and estimated to yield between 475,000 and 500,000 cumulative infections. Policy descriptions are available in Table 1 in the main text.

Index	PCR Per Day Multiplier	Antigen Per Day Multiplier	Contact Tracing Capacity	Booster Threshold	Vaccine Threshold	Mask Adherence	Mask Duration (Contact Traced)	Symp Testing OR	Quar Testing OR	Quar Adherence (Contact Traced)
1	4.28	9.19	10,970	0.49	0.38	0.04	2.94	87.79	93.04	0.98
2	2.61	7.53	22,879	0.31	0.09	0.07	0.75	19.66	86.65	0.71
3	2.59	7.86	9,566	0.02	0.72	0.03	0.36	97.32	75.17	0.7
4	5.51	1.29	6,904	0.08	0.73	0.05	10.76	28.19	89.81	1
5	4.97	9.81	19,023	0.05	0.47	0.06	3.98	22.82	9.85	1
6	8.89	1.08	31,999	0.06	0.02	0.02	2.95	63.15	88	0.93
7	4.44	1.25	11,529	0.45	0.04	0.09	4.22	79.57	7.57	0.71
8	8.5	9.58	13,556	0.47	0.05	0	12.53	30.44	24.92	0.89
9	1.26	1.6	18,422	0.01	0.58	0.02	13.68	68.22	10.02	0.77
10	7.58	8.64	32,878	0.04	0.07	0.03	6.48	95.04	9.35	0.77
Range	[1,10]	[1,10]	[6k,60k]	[0.0,0.5]	[0.0,0.75]	[0,0.2]	[0,14]	[10,100]	[1,100]	[0.7,0.9]

Table S3: Summary of experiments and their research goal.

Experiment	# of Simulations	Rationale
Calibration	< 500	Done iteratively while building model and conducting initial tests
LHS Sample (Figs. 2, 3, 4)	30,000 (1,500 policies, replicated 20 times)	Seeking at least 100 points per policy dimension, plus the maximum strength of each policy
LHS Validation	500	Validation of global emulator model, summarized via RMSE between simulator and emulator output
IMSPE Sensitivity	15,000	Conducted to assess feasibility of building up design space sequentially, selected via Integrated Mean-Squared Prediction Error (IMSPE)
Proposed Policies	200 (10 policies, replicated 20 times)	Seeking a candidate set of policies that meet the policy goal (~500k cumulative infections) and are "small" (ranked by the norm of the policy vector)
Proposed Policies (Maximin)	200 (10 policies, replicated 20 times)	Sensitivity to Proposed Policies, prioritizes policies being maximally different from one another while still meeting the policy goal of ~500k cumulative infections
Proposed Policies (Validation)	500	Validation of simulator and emulator dynamics in region of policy interest (~500k cumulative infections), summarized via RMSE
Proposed Policies (Expected Improvement)	200	Conducted to assess feasibility of directly targeting high-performing policies, as measured via: $$f(x) = (Y-5)^2 + \|\|x\|\|_2$$ Where Y is cumulative infections (in 100k) and x is a ten-dimensional policy vector on the 0-1 scale
